# Roles and action mechanisms of herbs added to the emulsion on its lipid oxidation

**DOI:** 10.1007/s10068-020-00800-z

**Published:** 2020-07-25

**Authors:** Eunok Choe

**Affiliations:** Department of Food and Nutrition, Inha University, 100 Inha-ro, Michuhol-gu, Incheon, 22212 Republic of Korea

**Keywords:** Herb, Polyphenol, Lipid oxidation of emulsion, Interaction, Tocopherol

## Abstract

Quality of food emulsions is mainly determined by their physicochemical stability such as lipid oxidation, and herbs as antioxidative food materials are added to improve their quality and shelf-life. Despite the extensive researches, the chemistry and implications of herb addition in the lipid oxidation of emulsions are still confusing. This review intended to provide the information on the roles and action mechanisms of herbs in the lipid oxidation of food emulsions, with focuses on polyphenols. Polyphenols act as antioxidants mainly via reactive oxygen species scavenging and metal chelating; however, their oxidation products and reducing capacity to more reactive metal ions increase the lipid oxidation. Factors such as structure, concentration, and distribution determine their anti- or prooxidant role. Interactions, synergism and antagonism, among polyphenol compounds and the effects of tocopherols derived from oil on the antioxidant activity of herbs were also described with the involving action mechanisms.

## Introduction

Many foods exist as emulsions which are mixtures of two or more liquids, such as oil and water, that are normally immiscible. Food emulsions include homogenized milk, butter, salad dressing, mayonnaise, and ice cream. An oil-in-water (O/W) emulsion consists of oil droplets dispersed in an aqueous phase; however, a system of water as dispersed (internal) phase and oil as continuous (external) phase forms water-in-oil (W/O) emulsion (McClements, 2008). Multiphase emulsions such as O/W/O and W/O/W are also prepared to protect or release specific ingredients, or produce low-fat foods (Fumiaki et al., 2008; Ito et al., 2012; Serdaroglu et al., 2015; Yoshida et al., 1999). The aqueous phase may contain water-soluble ingredients including sugars and salts, and oil phase may contain a variety of lipid-soluble components such as di- and monoacylglycerols, fatty acids, and tocopherols, whereas surface-active components including phospholipids are distributed in the interfacial region (McClements, 2008). Phospholipids as emulsifiers adsorb at the oil–water interface during the homogenization to prevent phase separation (Laguerre et al., 2017). These components, even at low concentrations, profoundly influence the quality of food emulsions during processing and storage (Traynor et al., 2013).

Quality and shelf-life of food emulsions are determined by their physical and chemical stability referring to an ability to resist changes in spatial distribution and in chemical structure, respectively, of ingredients over time (Mirhosseini et al., 2007). Lipid oxidation is one of the most important chemical reactions determining the chemical stability of food emulsions (Jacobsen, 2016), and many studies to reduce the lipid oxidation have been reported. Addition of antioxidants is the most common way to improve the oxidative stability of lipids in food emulsions, and herb extracts which contain high amounts of polyphenols have received a lot of attention, especially in O/W emulsions due to their hydrophilicity. Although there are many reports on the effects of herb extracts on the lipid oxidation (Abdalla and Roozen, 1999; Boroski et al., 2018; Frankel et al., 1996; Pawar et al., 2014), not many references have been found on the lipid oxidation in O/W emulsions. In addition, the chemistry and implications are still somewhat confusing since herb extracts have various polyphenols with different structures, chemistry, and functional properties. This review intended to provide more clear information on the role and action mechanism of herbs, especially focused on polyphenols, related to the lipid oxidation in food emulsions to help establish new directions for future research and application. Interactions among herbs/polyphenols and between herbs/polyphenols and tocopherols which are naturally derived from edible oil were also discussed.

### Lipid oxidation in food emulsions

Lipid oxidation in food emulsions occurs basically with the same mechanism as in bulk oils (Berton-Carabin et al., 2014), mostly free radical chain reaction including initiation, propagation, and termination steps as follows (Choe and Min, 2006):

$$\begin{array}{*{20}l} {\text{Initiation}} \hfill & {\quad \quad {\text{RH}} \to {\text{R}}^{\cdot} + {\text{H}}^{\cdot} } \hfill \\ {\text{Propagation}} \hfill & {\quad \quad {\text{R}}^{\cdot} +^{3} {\text{O}}_{2} \to {\text{ROO}}^{\cdot} } \hfill \\ {} \hfill & {\quad \quad {\text{ROO}}^{\cdot} + {\text{R}}^{\prime } {\text{H }} \to {\text{ROOH}} + {\text{R}}^{\prime \cdot} } \hfill \\ {\text{Termination}} \hfill & {\quad \quad {\text{ROO}}^{\cdot} + {\text{R}}^{\prime \cdot} \to {\text{ROOR}}^{\prime } } \hfill \\ {} \hfill & {\quad \quad {\text{R}}^{\cdot} + {\text{R}}^{\prime \cdot} \to {\text{RR}}^{\prime } } \hfill \\ \end{array}$$

Triacylglycerol (RH) molecules in oil should take a radical form (R·) in the initiation step by hydrogen abstraction by heat, metals, and/or light, and R· reacts with atmospheric triplet oxygen (^3^O_2_) to form peroxyl radical (ROO·). The peroxyl radicals abstract hydrogens from another triacylglycerol (R’H) molecules to produce hydroperoxides (ROOH) and another lipid (alkyl) radicals (R’·). Hydroperoxides are relatively stable at room temperature, but they are decomposed in the presence of heat, light, or metals, and produce low molecular-weight hydrocarbons and carbonyl compounds such as alcohols, aldehydes, and carboxylic acid, by hemolytic scission (Choe and Min, 2006). All radicals produced during the lipid oxidation themselves catalyze the oxidation reaction, especially in the propagation step. When the radicals react each other, non-radical compounds (ROOR’, RR’) are resulted and there is no more participation in the reaction cycle in the termination step (Choe and Min, 2006).

The lipid oxidation of food emulsions occurs mostly at the water–oil interface rather than the air-oil interface, and is governed by the critical micelle concentration of hydroperoxides and its modification by other amphiphilic compounds (Budilarto and Kamal-Eldin, 2015). The nature of the interface determines the contact among reactants such as oxygen, oil, and metal ions, and thus diffusion is more important in controlling lipid oxidation of the emulsion since it is related with mobility of triacylglycerol molecules inside the oil droplets (Berton-Carabin et al., 2014). Hydroperoxides and free radicals are usually present on the surface of oil droplets and can react with other triacylglycerol molecules, and therefore, the rate of lipid oxidation depends on the speed of these accelerators (hydroperoxides and free radicals) to diffuse within a droplet from one region to another (McClements and Decker, 2000). When surface-active lipid hydroperoxides are accumulated beyond their critical micelle concentration during lipid oxidation, the mechanism for mass transport shifts from the slow transfer of collision-exchange-separation pathway to the fast micelle-assisted mechanism, which may be the transition from the initiation to the propagation step (Laguerre et al., 2017). Under these circumstances, the transfer mechanism for antioxidants changes from diffusion to collision-exchange-separation (Laguerre et al., 2017). Amphiphilic compounds in the O/W emulsion arrange themselves to position the polar head groups at the surface and non-polar tails in the interior (Hazahari et al., 2018). Amphiphilic compounds exceeding the critical micelle concentration protect the lipids against oxidation by diluting the substrate or perhaps by replacing lipids at the interface, making it less accessible to radical attack (Ponginebbi et al., 1999). Soy-derived phosphatidylcholine and phosphatidylethanolamine reduced the iron-catalyzed autoxidation of water-in-canola oil (1:1, w/w) emulsion via a physical barrier to oxygen as well as hydrogen donation (Choe and Choe, 2016). The phosphatidylcholine at 250 mg/kg decreased the photosensitized lipid oxidation of water-in–sunflower oil emulsion (1:1, w/w) by chemical quenching of singlet oxygen (Lee and Choe, 2011).

There are contrasting reports on the difference in the lipid oxidation rates between emulsions and bulk oils. Lipid oxidation may be more favored in the emulsions than in bulk oils due to the interface which increases contact between oil and prooxidative metal ions or dissolved oxygen in the aqueous phase (Cuvelier et al., 2003; Frankel et al., 2002; Nikovska, 2012). Incorporation of oxygen during homogenization for emulsification was reported to contribute to faster oxidation in emulsions than in bulk oils (Horn et al., 2012). On the other hand, some studies reported no difference (Khan and Shahidi, 2000; McClements and Decker, 2000) and even lower lipid oxidation in the emulsions than in bulk oils (Belhaj et al., 2010). This was suggested to be attributed to slower diffusion of triacylglycerol molecules in the core of oil droplets in the emulsions (10^−8^ cm^2^/s order) than in bulk oils (10^−7^ order) (Hennere et al., 2009a; 2009b; Kabri et al., 2013).

Previous reports on the mechanism of lipid oxidation in the emulsions paid attention to transition metal ions in the aqueous phase and the hydroperoxides on the surface of the oil droplets. It is well-known that transition metal (iron and copper) ions promote the lipid oxidation by producing radicals. These metal ions in emulsions can be derived from oil and water. Refined soybean oil contains iron and copper at 200 and 2.5 μg/kg (Sleeter, 1981), respectively. Iron and copper levels in drinking-water were reported as < 0.3 mg/L (WHO, 2003) and in the range from ≤ 0.005 to > 30 mg/L (WHO, 2004), respectively. Metal ions react directly with triacylglycerol molecules in oil to produce lipid (alkyl) radicals, decompose hydroperoxides to alkoxyl (RO·) and peroxyl radicals, and produce reactive oxygen species such as superoxide anion radicals (O_2_·^−^), singlet oxygen (^1^O_2_), and hydroxyl radicals (HO·) as follows (Choe and Min, 2006), and all of which accelerate the lipid oxidation.

$${\text{Fe}}^{3 + } \,\left( {{\text{or}}\,{\text{Cu}}^{2 + } } \right) + {\text{RH}} \to {\text{Fe}}^{2 + } \,\left( {{\text{or}}\,{\text{Cu}}^{ + } } \right) + {\text{R}}^{\cdot} + {\text{H}}^{ + }$$

$${\text{ROOH}} + {\text{Fe}}^{2 + } \,\left( {{\text{or}}\,{\text{Cu}}^{ + } } \right) \to {\text{RO}}^{\cdot} + {\text{Fe}}^{3 + } \,\left( {{\text{or}}\,{\text{Cu}}^{2 + } } \right) + {\text{OH}}^{ - }$$

$${\text{ROOH}} + {\text{Fe}}^{3 + } \,\left( {{\text{or}}\,{\text{Cu}}^{2 + } } \right) \to {\text{ROO}}^{\cdot} + {\text{Fe}}^{2 + } \,\left( {{\text{or}}\,{\text{Cu}}^{ + } } \right) + {\text{H}}^{ + }$$

$${\text{Fe}}^{2 + } \,\left( {{\text{or}}\,{\text{Cu}}^{ + } } \right) +^{3} {\text{O}}_{2}^{{}} \to {\text{Fe}}^{3 + } \,\left( {{\text{or}}\,{\text{Cu}}^{2 + } } \right) + {\text{O}}_{2}^{\cdot - }$$

$${\text{O}}_{2}^{\cdot - } + {\text{O}}_{2}^{\cdot - } + 2{\text{H}}^{ + } \to^{1} {\text{O}}_{2} + {\text{H}}_{2} {\text{O}}_{2}$$

$${\text{H}}_{2} {\text{O}}_{2} + {\text{O}}_{2}^{\cdot - } \to {\text{HO}}^{\cdot} + {\text{OH}}^{ - } +^{1} {\text{O}}_{2}$$

### Herb effects on the lipid oxidation of emulsions

Herbs generally refer to the leafy green or flowering parts of fresh or dried plants valued for their medicinal and savory or aromatic properties, and have been used for flavoring and garnishing foods (Peter and Shylaja, 2004). Basil (*Ocimum basilicum*), oregano (*Origanum vulgare*), peppermint (*Mentha piperita*), rosemary *(Rosmarinus officinalis* L.), and thyme (*Thymus vulgaris*) are typical examples of herbs, and many researches on their effects on the lipid oxidation of emulsions have been reported. Rosemary extracts have proven to be effective for controlling lipid oxidation in the corn oil-in-water emulsion (Frankel et al., 1996), and 10% fish oil-in-water emulsion at 4000 mg/kg (Erdmann et al., 2015). Lipid oxidative stability of 20% sunflower oil-in-water emulsion was significantly improved by the addition of sage extract (600 and 1200 mg/kg) whose antioxidant activity was higher than that of BHT at 300 mg/kg (Abdalla and Roozen, 1999). Rosemary or thyme extract at 100 mg/kg decreased the hydroperoxide production in 40% soybean oil-in-water emulsion (Kim and Choe, 2016). Basil or peppermint extract significantly reduced the production of secondary oxidation products of lipids as well as hydroperoxides in 40% sunflower oil-in-water emulsion at 100 mg/kg (Kim and Choe, 2016; Kim et al., 2017) and 25% olive oil-in-water emulsion (Mihov et al., 2012). Lavender (*Lavandula angustifolia*) and thyme extracts showed higher antioxidant activity than rosemary extracts in the O/W emulsion (Gallego et al., 2013). The antioxidant activity of the peppermint or basil extract at 400 mg/kg was higher than that of rosemary, oregano, or thyme extract in the 40% soybean oil-in-water emulsions, and the antioxidant activity showed a concentration dependence within a range between 0 and 400 mg/kg (Kim and Choe, 2016).

The antioxidant activity derived from herb extracts was different depending on the herb and the extracting solvents; non-polar extracts of barley with 80% acetone showed higher reducing power than those with 80% ethanol or methanol and water extracts, whereas the reverse phenomena were observed in hydroxyl or superoxide anion radical scavenging activity and metal chelating activity (Zhao et al., 2006). While the methanol extract of parsley (*Petroselinum crispum*) at 1000 mg/kg acted as antioxidant in the lipid oxidation of the O/W emulsion, water extract acted as prooxidant. Both the methanol and water extracts of cilantro (*Coriandrum sativum*) showed prooxidant activity (Wong and Kitts, 2006). Water extract of lemon balm (*Melisa officinalis*) improved the lipid oxidative stability of olive oil-in-water emulsion at 477 mg/kg corresponding to the antioxidant activity of BHA at 200 mg/kg (Poyato et al., 2013). Different antioxidant activity of herbs depending upon the extracting solvents is mainly due to the chemical composition of the extract. Herb extracts mostly contain polyphenols, with less amount of other minor compounds such as tocopherols, carotenoids, and chlorophylls, and non-polar solvents tend to result in less polar components in the extracts. The 75% ethanol extracts of basil contained 58.3, 3.42, 2 (3.25), and 3.21 g/kg of polyphenols, chlorophylls, carotenoids, and tocopherols, respectively (Kim et al., 2017). Contents of polyphenols, chlorophylls, carotenoids, and tocopherols in the 75% ethanol extract of peppermint were 169, 4.40, 0.52, and 1.32 g/kg, respectively (Lee and Choe, 2018). Among these compounds, a main contributor to the reduced lipid oxidation of the O/W emulsions was high contents of polyphenols, and tocopherols were also very important antioxidant despite low levels; on the other hand, carotenoids and chlorophylls tended to increase the lipid oxidation even in the dark, possibly due to their oxidation products (Lee and Choe, 2018). They suggested that polyphenols could be the first consideration in the herb extracts relating to the lipid oxidation of O/W emulsions as antioxidants.

### Polyphenol composition of herbs

Polyphenols are generally defined as compounds having more than 12 phenolic hydroxyl groups (Haslama and Cai, 1994); however, the definition is generally extended to the compounds exclusively derived from the phenylpropanoid pathway with more than one phenolic unit and deprived of nitrogen-based functions (Bravo, 1998). The phenylpropanoids are a diverse family of organic compounds that are synthesized by plants from phenylalanine and tyrosine. The name is derived from the six-carbon, aromatic phenyl group and the three-carbon propene tail of coumaric acid (Barros et al., 2016; Navarre et al., 2016). Polyphenols protect the plants from environmental stresses such as pathogen attack, UV-irradiation, high temperature/light, wounding, nutrient deficiencies, and herbicide treatment (Solecka, 1997).

The food industry has utilized herbs mostly as flavorings, antioxidants, and preservatives for existing food processing techniques (Giacometti et al., 2018), and polyphenols are the most important in performing these functions. Rosmarinic acid is one of the polyphenol compounds which are the most commonly found in herbs, especially thyme, mugwort (*Artemisia vulgaris*), peppermint, and basil (Chamila et al., 2003; Kim and Choe 2016; Loughrin and Kasperbauer, 2001; Shan et al., 2005; Zheng and Wang, 2001). The 80% methanol extract of spearmint (*Mentha spicata*) is composed of rosmarinic acid and its derivatives (230.5 ± 13.5 mg/g) with smaller amounts of salvianolic, caffeoylquinic, hydroxybenzoic, and hydroxycinnamic acids, flavones, and flavanones (Cirlini et al., 2016). Polyphenol composition varies depending on the kind and species of herbs and the polarity of extracting solvents (Dent et al., 2013). This is one of the reasons why studies on the effects of herb extracts on the lipid oxidation of emulsions have reported different behaviors, and the information on the polyphenol composition of herb extracts should be provided in evaluating their anti- or prooxidant activity. The methanol extract of apple mint (*Mentha suaveolens*) and peppermint contained high amount of quercetin (9.5 and 5.1 mg/g, respectively); however, rutin was abundant in horse mint (*Mentha longifolia*) and spearmint extract (11.7 and 3.1 mg/g, respectively). Other compounds such as chlorogenic, caffeic, coumaric, ferulic, cinnamic, and rosmarinic acids and epicatechin were present at less than 1 mg/g in these extracts (Park et al., 2019).

Wojdyło et al. (2007) reported caffeic acid as a major phenolic acid in 80% methanol extracts of lemon balm, oregano, mugwort, rosemary, and thyme at 8.58, 6.49, 3.04, 4.06, and 5.17 mg/g, respectively. Quercetin in sage (*Salvia officinalis*) extract (1.78 mg/g) and luteolin in rosemary extract (6.16 mg/g) were major flavonoids; however, no flavonoids were detected in oregano, lemon balm, mugwort, and thyme extracts (Wojdyło et al., 2007). Catechin was detected in the 80% ethanol extracts of rosemary, basil, and peppermint, but much less amount than rosmarinic acid (Kim and Choe, 2016). Isosalvianolic acid A (24.21%), salvianolic acid B, danshensu, luteolin-rutinoside, and luteolin in addition to rosmarinic acid (35.94%) were detected in the 80% ethanol extract of peppermint (Guedon and Pasquier, 1994; Hadjmohammadi et al., 2013; Kim and Choe, 2018).

The polarity of extracting solvents also affects polyphenol composition of the herb extracts. Dent et al. (2013) reported a close relationship of the extraction efficiency of polyphenols with the polarity of solvents; in extracting polyphenols from sage, binary solvent systems of ethanol in water (30%) were more efficient than mono-solvent system (100% ethanol or water). Carnosic acid and carnosol were predominant in the methanol or acetone extract of rosemary (467 and 325 g/kg), while they were not found in the water extract (Moreno et al., 2006). Reports on the polyphenol extraction from herbs strongly have suggested that selection of extracting solvents can be very crucial to determine the antioxidant activity of herb extracts in O/W emulsions. Table 1 provides the list of polyphenol compounds detected in some of herbs, depending on the extracting solvents.

**Table 1:** Polyphenol composition of some herb extracts with different solvents

Herbs	Extracting solvent	Polyphenols	References
Oregano (*Origanum dictamus*)	Water	Chlorogenic acid, rutin, luteolin-7-*O*-glucoside, apigenin-7-*O*-glucoisde, rosmarinic acid, luteolin	Kaliora et al. (2014)
	62.5% Methanol	Vanillic acid, protocatechuic acid, syringic acid, gallic acid, cinnamic acid, *o*-coumaric acid, *p*-coumaric acid, caffeic acid, chlorogenic acid, rosmarinic acid, chrysin, epicatechin, naringenin, catechin, genistein, quercetin	Proestos and Komaitis (2013)
Oregano (*O. majoram* L.)	Water	Vanillic acid, protocatechuic acid, syringic acid, gallic acid, cinnamic acid, *o*-coumaric acid, *p*-coumaric acid, ferulic acid, caffeic acid, sinapic acid, rosmarinic acid, chrysin, epicatechin, naringenin, catechin, kaempferol, quercetin	Kaliora et al. (2014)
Oregano (*O. vulgare*)	50% Ethanol	Rosmarinic acid, caffeic acid, protocatechuic acid, *p*-coumaric acid, *p*-hydroxybenzoic acid, syringic acid	Vallverdú-Queralt et al. (2014)
Dill (*Anethum graveolens)*	50% Ethanol	Chlorogenic acid, *p*-hydroxybenzoic acid, caffeic acid, protocatechuic acid, ferulic acid, *p*-coumaric acid, rosmarinic acid, syringic acid	Vallverdú-Queralt et al. (2015)
Rosemary *(Rosmarinus officinalis* L.)	80% Ethanol	Rutin, catechin, quercetin, gallic acid, ferulic acid, *p*-coumaric acid, *p*-cinnamic acid, kaempferol	Pereira et al. (2017)
	50% Ethanol	Rosmarinic acid, *p*-hydroxybenzoic acid, caffeic acid, protocatechuic acid, *p*-coumaric acid, syringic acid, ferulic acid, quercetin, chlorogenic acid	Vallverdú-Queralt et al. (2014)
	Methanol	Luteolin, caffeic acid, apigenin, ferulic acid	Wojdyło et al. (2007)
	Acetonitrile with formic acid (2%)	Carnosic acid, carnosol, rosmanol, hispidulin-rutinoside, apigenin, hesperidin, rosmarinic acid, luteolin,	Mena et al. (2016)
Thyme (*Thymus vulgaris*)	50% Ethanol	Rosmarinic acid, caffeic acid, *p*-hydroxybenzoic acid, protocatechuic acid	Vallverdú-Queralt et al. (2014)
	Methanol	Caffeic acid, ferulic acid	Wojdyło et al. (2007)
Peppermint (*Mentha piperita*)	Acetonitrile	Eriocitrin, rosmarinic acid, luteolin-7-O-glucoside, hesperidin, eriodictyol, isorhoifolin, caffeic acid, luteolin	Dorman et al. (2009)
	Methanol	Rosmarinic acid, eriocitrin, luteolin-7-O-glucoside, hesperidin, isorhoifolin, luteolin	Dorman et al. (2009)
	Water	Rosmarinic acid, luteolin-7-O-glucoside, eriocitrin	Dorman et al. (2009)
	Methanol-ethanol (1:1)	Chlorogenic acid, rutin, caffeic acid, 4-hydroxybenzoic acid, ferulic acid, vanillic acid	Farnad et al. (2014)
Sage *(Salvia officinalis)*	Water	Apigenin, luteolin-7-O-glucuronide, rosmarinic acid, salvianolic acid, sagerinic acid, scuttelarein, saponarin	Afonso et al. (2019), Walch et al. (2011)
	Methanol	Rosmarinic acid, carnosic acid, salvianolic acid B and A, caffeic acid	Fotovvat et al. (2018)

### Roles of polyphenols in the lipid oxidation of emulsions

Polyphenols have been reported to have antioxidant activity via reactive oxygen species scavenging and/or metal chelating (Choe and Min, 2009). A polar paradox hypothesis on the antioxidant activity of antioxidants focuses the interfacial phenomena, and states that more polar polyphenols are more efficient antioxidants than nonpolar tocopherols in nonpolar systems such as bulk oils (Frankel et al., 1994; Porter et al., 1989). Nonpolar antioxidants are more effective in relatively more polar media, such as O/W emulsions or liposomes (Shahidi and Zhong, 2011). However, other studies particularly with real food systems (mostly O/W emulsions) showed no satisfaction with the polar paradox; polyphenols produce free radicals to accelerate lipid oxidation in emulsions depending on the reaction systems (Eghbaliferiz and Iranshahi, 2016; Zhou and Elias, 2012). Factors to change the role of polyphenols, anti- or prooxidant, in the emulsions include emulsifiers, pH, metals, metal chelators, emulsifiers, and temperature (Medina et al., 2012). For example, (−)-epigallocatechin-3-gallate acted as antioxidant in the flaxseed oil-in-water emulsion at pH 7; however, it was a prooxidant at pH 3 (Zhou and Elias, 2012). The antioxidant activity of caffeic acid was much lower in the Citrem (citric acid ester of mono- and diacylglycerol) stabilized fish oil-in-water emulsion than in the Tween 80 stabilized emulsion and even showed prooxidant activity (Sørensen et al., 2017). Physicochemical characteristics of antioxidants such as polarity may determine their antioxidant activity; propyl gallate which is non-polar is concentrated at the oil–water interface in the emulsion and preferentially donates hydrogen to lipid radicals rather than metal chelation in the aqueous phase (Aaneby, 2012). Different roles of the same polyphenol compound under specific conditions have been explained with diverse mechanisms, which is described in the following sections.

#### As antioxidants

**Fig. 1:**
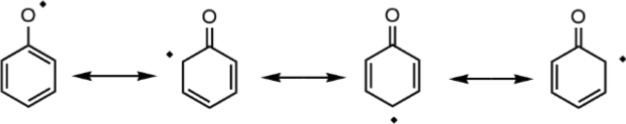
Resonance structures of phenoxyl radical

The antioxidant activity of polyphenols is resulted from scavenging reactive oxygen species including hydroxyl, peroxyl and alkyl radicals, chelating prooxidative metals, and decomposing lipid peroxides (García-Pérez et al., 2018; Kurek-Górecka et al., 2014). Hydroxytyrosol and oleuropein improved the oxidative stability of lipids in an O/W emulsion through a complex formation with prooxidative copper in addition to scavenging radicals (Paiva-Martins et al., 2006). Polyphenols contain one or more aromatic rings bearing at least two hydroxyl groups in their structures, and thus can easily donate phenolic hydrogen to radicals, which is related to the reduction potential. Reduction potentials of 3,5-dihydroxyanisol (0.84 V), 2,4-dihydroxyacetophenone (0.89 V), 2-methoxy-4-methylphenol (0.68 V), catechol (0.53 V), 4-methylcatechol (0.52 V), and methyl gallate (0.56 V) radicals were lower than that of alkyl and peroxyl radicals of lipids (1.06 V) (Jovanovic et al., 1996). Although phenoxyl radicals (PO·) from polyphenols (POH) are produced, their resonance structures (Fig. 1) enable them to be more stable than alkyl, peroxyl, or alkoxyl radicals of lipids produced during lipid oxidation. When phenoxyl radicals combine together to form dimers (POOP), the chain reaction can be terminated (Mahoney and Weiner, 1972). Some of polyphenol dimers even show enhanced antioxidant activity; the superoxide anion radical scavenging capacity of ferulic acid dimers were higher at pH 7.4 than that of ferulic acid, and the order of scavenging activity was 5-5-diferulic acid > 8-*O*-4-diferulic acid > 8-8-diferulic acid (Cano et al., 2002).

$${\text{ROO}}^{ \cdot } + {\text{POH}} \to {\text{ROOH}} + {\text{PO}}^{ \cdot }$$

$${\text{R}}^{ \cdot } + {\text{POH}} \to {\text{RH}} + {\text{PO}}^{ \cdot }$$

$${\text{RO}}^{ \cdot } \, + {\text{POH}} \to {\text{ROH}} + {\text{PO}}^{ \cdot }$$

$${\text{PO}}^{ \cdot } \, + {\text{PO}}^{ \cdot } \, \to {\text{POOP}}$$

**Fig. 2:**
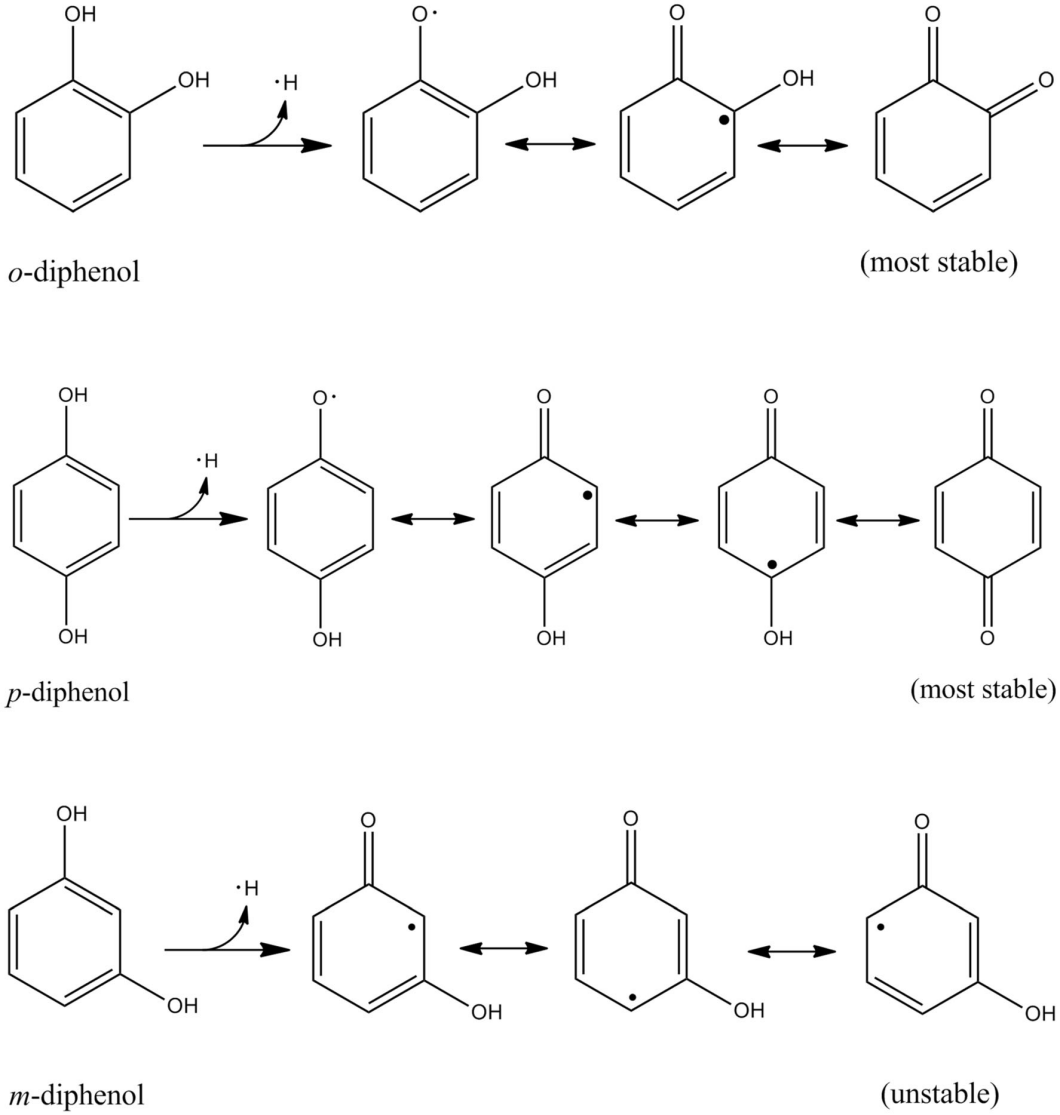
Resonance structures of diphenol upon hydrogen abstraction

Radical scavenging activities of polyphenols depend on the chemical structure and distribution in each phase of an emulsion, the oil, interfacial, and aqueous regions (García-Pérez et al., 2018). Since polyphenols produce phenoxyl radicals upon hydrogen donation to scavenge various lipid radicals, the stability of the phenoxyl radicals determine the efficiency of polyphenols as antioxidants; the higher the stability of the phenoxyl radical is, the more efficient the polyphenol is. The phenoxyl radical can be stabilized by intramolecular hydrogen bonds and the extended delocalization and conjugation of the electrons enhanced by resonance phenomena (Chen et al., 2015). Polyphenols with low ionization potential and O–H bond dissociation energy are expected to have high radical scavenging activity (Al-Sehemi and Irfan, 2017). The phenoxyl radical is further stabilized by substitution with bulky *o*-substituents, coordination to metal ions or *p*-conjugation expansion (Aotake et al., 2015). Phenoxyl radicals having hydroxyl groups at *o*- and/or *p*-position derived from diphenols are more stable than those at *m*-position as shown in Fig. 2. In addition to the substituent position, kinds of substituent groups in the benzene ring affect the radical scavenging activity of polyphenols. The electron-withdrawing groups increase the ionization potential and O–H bond dissociation energy, and thus decrease the radical scavenging activity (Zheng et al., 2018). The electron-donating substituents show an opposite behavior. Catechol (*o*-hydroxyphenol) reacts with lipid radicals faster than *o*-methoxyphenols (O-methylated catechol) due to higher stability of their semiquinone radicals upon hydrogen donation (Aaneby, 2012). The oxygen atom of the methoxyl and hydroxyl groups in the structure has lone pair electrons, and resulting p-π conjugation will increase the electron cloud density of the benzene ring, and will decrease the energy of π-π* electron transition (Gao et al., 2015). *o*-Methoxyphenols possess lower capacity for electron and hydrogen atom donation than catechols, which suggests higher radical scavenging activity of catechols. In addition, the formation of quinone upon a second electron donation becomes slow in *o*-methoxyphenols, and thus reduces the radical scavenging activity (Lemanska et al., 2004).

Lipid radical scavenging activities of polyphenols are higher when they are more distributed in the interfacial region (Losada-Barreiro et al., 2013a) at which lipid radical production occurs mostly (Freiría-Gándara et al., 2018). Polyphenols are predominantly located in the water-interfacial regions rather than in the oil droplet interior; meanwhile, more hydrophobic tocopherols are mostly located in the oil-interfacial regions (Lisete-Torres et al., 2012; Losada-Barreiro et al., 2013b). For the radical scavenging activity, the antioxidants should be transferred to the oil molecules, whose mechanism is affected by their hydrophobicity. Transfer of hydrophilic antioxidants from one oil droplet to another occurs mainly via diffusion through the water phase, while hydrophobic antioxidants are transferred by collision-exchange-separation process (Laguerre et al., 2017). Below a hydrophobicity threshold, the antioxidants are located in the water phase not close enough to the interfacial region, resulting in weaker antioxidant capacity. However, at and beyond the hydrophobicity threshold, the antioxidants are located at the interface and within the oil phase, respectively (Laguerre et al., 2017). The polarity of polyphenols is related with their solubility in the water phase, and affects their concentration at the interfacial region. Methyl carnosate (150–300 μM), whose partition coefficient for the oil phase to the water phase is 23.4 ± 2.3, was more located at the oil–water interfaces and in the oil phase, and was a more effective antioxidant than more polar carnosic acid (partition coefficient, 10.2 ± 1.1) in a corn oil-in-water emulsion (10:90, w/w) (Huang et al., 1996). Although the hydrophobicity of polyphenols affects their distribution in each phase of the emulsion, this is not always true; hydrophilic gallic or caffeic acid was more distributed in the interfacial region of emulsions than hydrophobic lauryl gallate, partly due to an emulsifier (Losada-Barreiro et al., 2014). They reported that an increase in the concentration of Tween 20 promoted the distribution of resveratrol in the interfacial region of the corn oil emulsions.

**Fig. 3:**
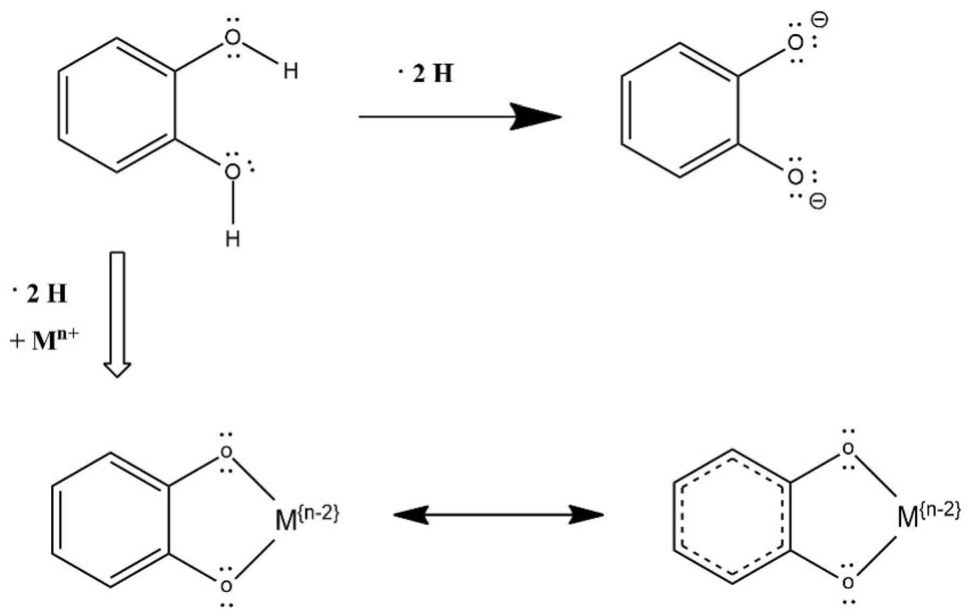
Mechanism for the catechol-metal complex formation (from Cass, 2014)

**Fig. 4:**
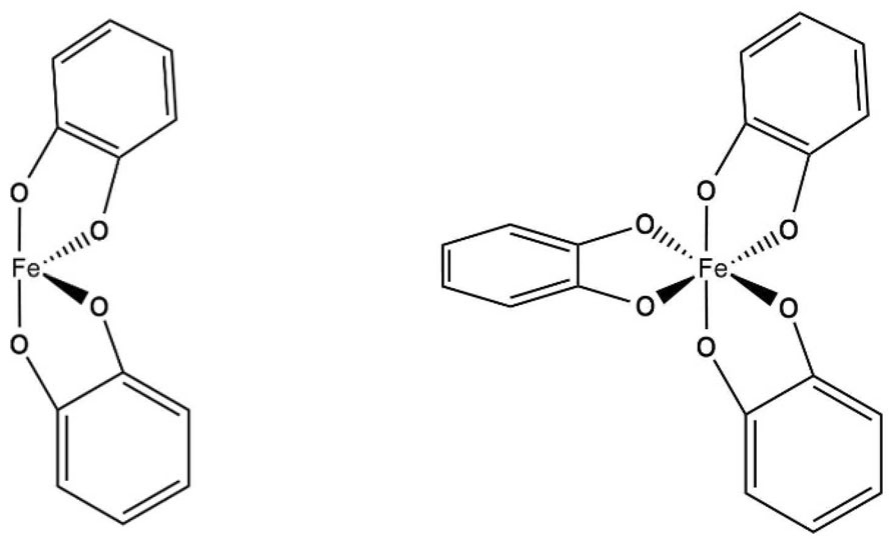
Examples of the coordination of two and three 2-ligands to iron (from Xu, 2013)

Antioxidant activity of polyphenols can be resulted from chelation of prooxidative metals. Ferrous (Fe^2+^), ferric (Fe^3+^), cuprous (Cu^+^), and cupric (Cu^2+^) ions are one of the leading causes to produce strong oxidants such as hydroxyl and peroxyl radicals (Kim and Choe, 2018). The Fe^2+^ is more active than Fe^3+^ in catalyzing the lipid autoxidation (Halliwell and Gutteridge, 2001; Mei et al., 1998). By chelating these metal ions, polyphenols help to reduce the formation and implication of the reactive oxygen species in the lipid oxidation of the emulsion. In addition, metal chelates (metal-polyphenol complex) are more prone to scavenge free radicals than the free polyphenols (Cherrak et al., 2016). Catechol or galloyl moieties in the structure of polyphenols are the responsible part for the complex formation with metal ions (Andjelkovica et al., 2006); double deprotonation in the hydroxyl group of catechol ligands leads to the binding of catechol to the Fe^3+^ (Fig. 3) (Cass, 2014). It was reported that ligands preferred a bridged bidentate mode of coordination (Pandey et al., 2019), and Xu (2013) suggested double (bis-) and triple (tris-) complex formation between catechol and iron (Fig. 4). The 3-hydroxy-4-keto group in flavonols and the 5,6,7-trihydroxyl group in flavones were reported to be the most efficient copper chelation sites in flavonoids (Ríha et al., 2014). The iron-chelating ability of protocatechuic acid, hydroxytyrosol, gallic acid, caffeic acid, and chlorogenic acid was reported to be 1.43, 2.66, 4.78, 8.12, and 20.13/M, respectively (Andjelkovića et al., 2006). The Fe^3+^ complex formation of epigallocatechin gallate and epicatechin gallate was lower than that of epigallocatechin and catechin due to the electron-withdrawing effect of the esters (Jovanovic et al., 1998). Complex formation of polyphenols with iron is dependent on their concentration and the pH (Kim and Choe, 2018), and caffeic acid needs to be usually at higher than 50 μM in vicinity of free radicals generated at the oil phase (Aaneby, 2012). Although caffeic acid having carboxyl group possesses a polar character, it becomes less polar at pH 3.5 due to protonation of the group, which may facilitate accessibility of caffeic acid at the interfacial region (Kristinová et al., 2009). Epigallocatechin gallate and gallic acid formed a complex with Cu^2+^, dominantly above neutral pH, which appreciably contributes to the metal speciation (Pirker et al., 2012).

#### As prooxidants

**Fig. 5:**
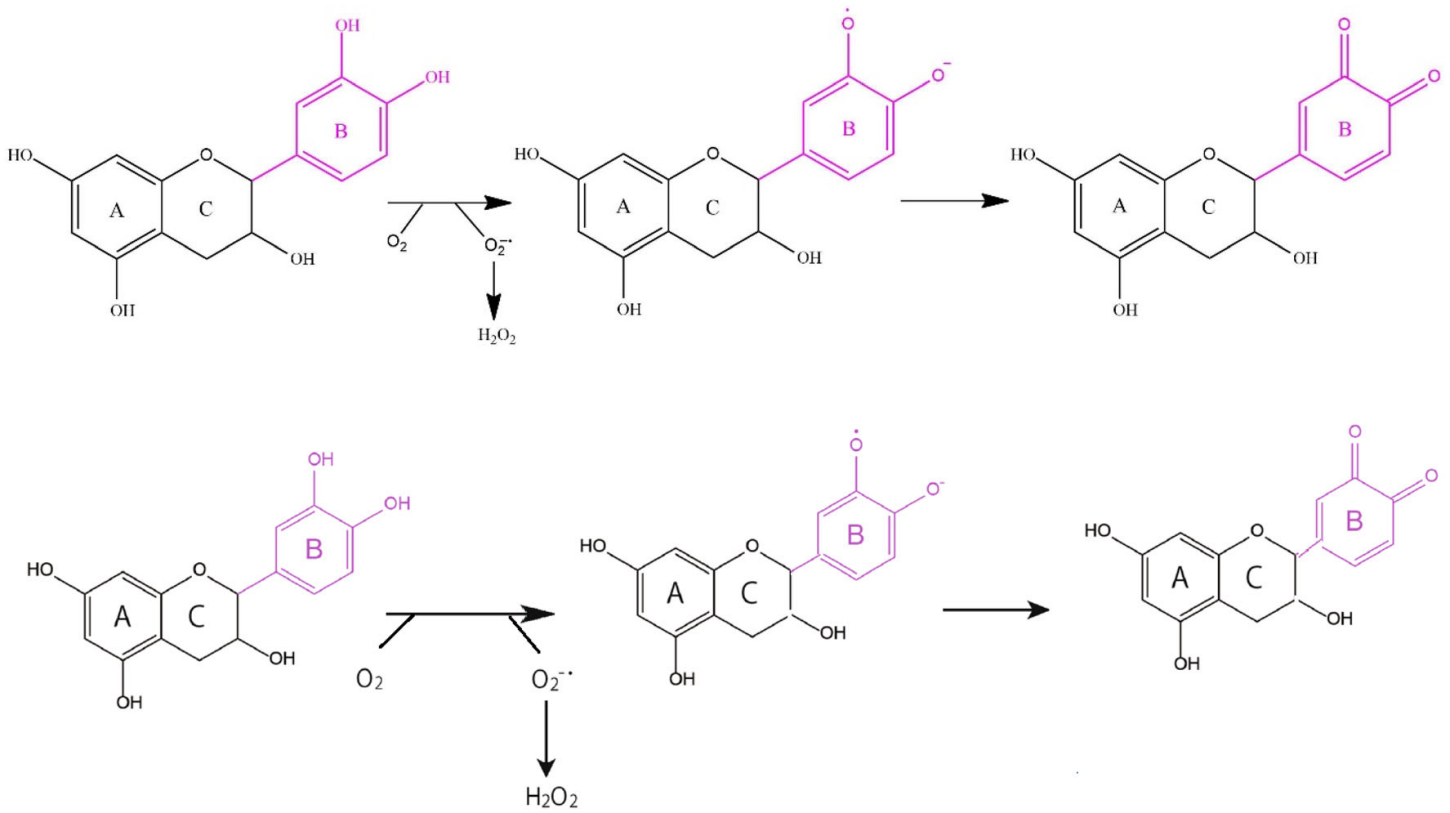
Oxidation of catechin and coupled generation of reactive oxygen species (modified from Shishido et al., 2018)

**Fig. 6:**
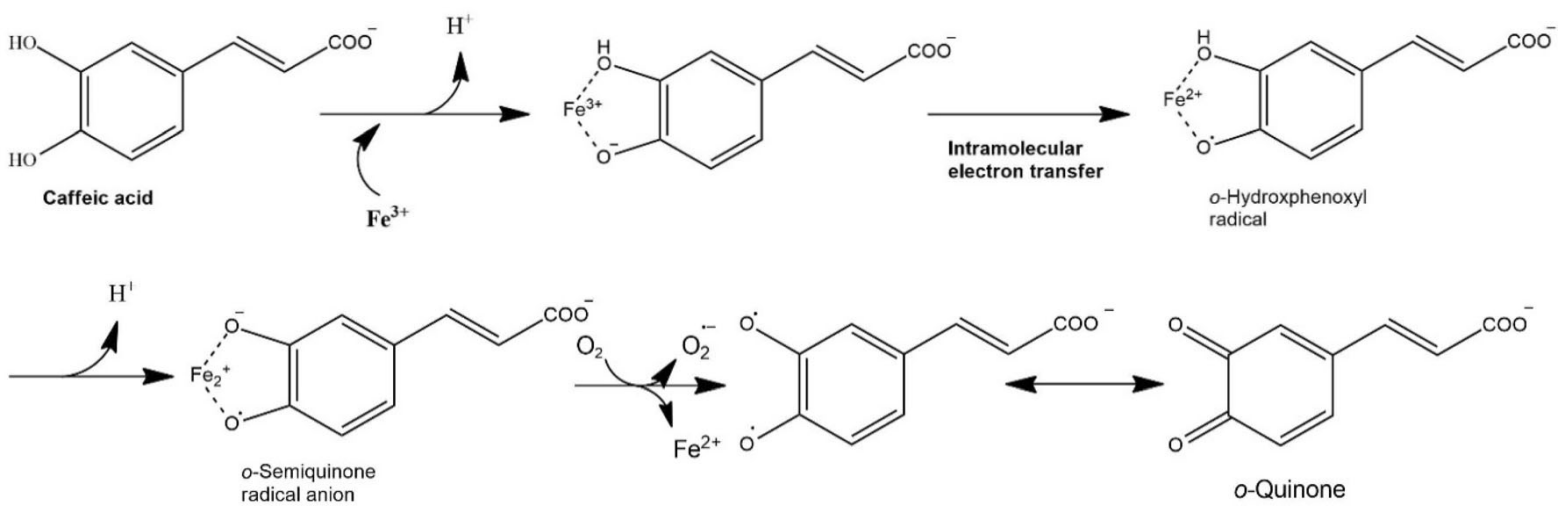
Mechanism for the reduction of Fe(III) to Fe(II) by caffeic acid (from Aaneby 2012; Kristinová et al., 2009)

In spite of the antioxidant activity of polyphenols, some of polyphenols show prooxidant activity under certain conditions such as at high concentration, and their interaction with metal ions can increase the lipid oxidation (Yordi et al., 2012). The prooxidant activities of polyphenols are derived from their autoxidation, in which semiquinone and superoxide anion radicals are produced. Polyphenols having either a pyrogallol or catechol structure such as quercetin and catechin can generate reactive oxygen species such as superoxide anion radicals, hydrogen peroxides, and peroxyl radicals as shown in Fig. 5 (Shishido et al., 2018). Pyrogallol-type flavonoids generate more hydrogen peroxides than the catechol-types (Miura et al., 1998). The prooxidant activities of polyphenols are also derived from the reduction of Fe^3+^ to more reactive Fe^2+^ (Zeraik et al., 2014). Gallic acid increased the hydroperoxide decomposition in a fish oil emulsion by reducing metal ions (Jacobsen et al., 2001). Figure 6 shows a complex formation of caffeic acid with Fe^3+^ which subsequently produces *o*-hydroxyphenoxyl radical through an intramolecular electron transfer reaction. The *o*-hydroxyphenoxyl radical is transformed to *o*-semiquinone anion radical upon hydrogen abstraction, and the *o*-semiquinone anion radical finally produces *o*-quinone upon release of Fe^2+^ (Aaneby, 2012; Kristinová et al., 2009).

The prooxidant activities of polyphenols are related with their redox potential; polyphenols with high oxidation potential (> 0.45 V) such as salicylic, hydroxybenzoic, vanillic, syringic, and coumaric acids, were reported to act as prooxidants at 4 mM (Simić et al., 2007). Besides their oxidizability, hydrophobicity can be a decisive factor; esterification of protocatechuic acid increased the prooxidant capacity (Zeraik et al., 2014). Concentration of polyphenols also affects the prooxidant activity; caffeic acid showed a maximum prooxidative effect at 25–50 μM in a herring oil-in-water (10:90, w/w) emulsion (Aaneby, 2012). Compared to many reports on the antioxidant activity of polyphenols, there are limited number of researches on the prooxidant activity and factors so far, which requires more research.

### Interactions among herbs/polyphenols in the lipid oxidation of emulsions

Polyphenols show different activities depending on their structure, concentration, and distribution as described previously, and the method to evaluate the antioxidant activity may also give different results (Sonam and Guleria, 2017). For this reason, it is not easy that the antioxidant activity of polyphenol mixture is predicted simply from that of pure compounds, and it is true for their interaction (Katalinic, 2015). It is also possible that one dose combination produces synergistic interactions while the another produces antagonism with the same substances (Pan et al., 2018; Sonam and Guleria, 2017).

Thyme showed a synergistic antioxidant activity with coriander (*Coriandrum sativum*) and celery (*Apium graveolens*) (Crespo et al., 2019), which can help to avoid undesirable side effects due to higher doses of one single herb (Jain et al., 2011). Synergism occurs when a stronger antioxidant is protected by a weaker one by regeneration or sacrificial oxidation and/or when 2 or more antioxidants with different antioxidant mechanisms are combined together (Choe and Min, 2009). Hot water extracts of green tea showed a synergistic interaction with clove basil (*Ocimum gratissimum*) to reduce lipid oxidation at the combination ratio of 1:1, 1:2, 1:3, 3:1, and 2:1 (Farooq and Sehgal, 2019). Synergistic interactions among polyphenols were shown; quercetin showed synergism with phloretin and epicatechin (1 μM each) in the Cu^2+^-mediated oxidation of human low density lipoproteins (Yeomans et al., 2005). Rosmarinic acid showed synergistic effects with quercetin and caffeic acid in the AAPH-induced oxidation of linoleic acid emulsions (Peyrat-Maillard et al., 2013). Synergistic antioxidant activity is dependent on the type of antioxidant and its concentration, and thus the appropriate concentration and combination of antioxidants may be important factors for the maximum synergism (Sonam and Guleria, 2017).

Antagonism has been known to arise by regeneration of the less effective antioxidant by the more effective one, oxidation of the more effective antioxidant by the less effective antioxidant radicals, competition between antioxidant radical adduct formation and the antioxidant regeneration, and alteration of microenvironment of one antioxidant by another (Choe and Min, 2009). Moderate antagonism was shown in the DPPH radical scavenging activity between hot water extracts of green tea and clove basil at the ratio of 1:2 (Farooq and Sehgal, 2019). Caffeic acid showed antagonistic effects with the (+)-catechin or quercetin in equimolar proportions in the AAPH-induced oxidation of linoleic acid emulsions (Peyrat-Maillard et al., 2013). Meanwhile, Olszowy et al. (2019) suggested that the antagonism caused by the change in radical neutralization ability of antioxidants could be due to the effect of different reaction kinetics between a given antioxidant and the ABTS cation radical. Therefore, it should be considered that antagonism may be derived from a reaction between polyphenols and radicals used in evaluating the antioxidant activity rather than mutual interaction between individual polyphenols.

### Effects of tocopherols on the antioxidant activity of herbs/polyphenols in the lipid oxidation of emulsions

The anti- or prooxidant activities of herb extracts or polyphenols depend on the oxidation conditions as described previously, and other components present in the emulsion such as proteins and antioxidants other than polyphenols also affect the activities. Polyphenols derived from the aqueous extract of roselle (*Hibiscus sabdariffa*) showed high protection against lipid oxidation of the whey spread emulsion via a dimer formation with whey proteins (Chikhoune et al., 2017). Rice glutelin enhanced the antioxidant activity of procyanidin via formation of molecular complexes primarily through hydrophobic attractive forces (Dai et al., 2019). The exposed sites of protein upon breaking its hydrogen bonds are important binding sites for polyphenols (Siebert et al., 1996).

Since interactions between polyphenols and protein or peptides are mostly limited to meat and fish products, tocopherols which are commonly derived from edible oils are more important concerning the interaction with polyphenols in food emulsions. Lipid oxidation of the soybean oil-in-water (4:6, w/w) emulsion with added ethanol extract of peppermint at 400 mg/kg was lower in the co-presence of γ- or δ-tocopherol at 600 mg/kg; however, an opposite result was shown in the emulsion with α-tocopherol (Kim and Choe, 2019). However, co-addition of rosemary extract and tocopherols in meat products did not increase the antioxidant efficacy of these individually used compounds (Resurreccion and Reynolds, 1990). Caffeic acid at 100 μM was very efficient antioxidant in fish oil-in-water emulsion (pH 7) in the presence of endogenous tocopherols, while it was a prooxidant without tocopherols (Sørensen et al., 2017). Quercetin at 1 μM showed a significant (*p* < 0.001) synergism with α-tocopherol at 5 μM; however, there was no interaction observed from epicatechin, hesperetin, or phloretin with α-tocopherol (Yeomans et al., 2005). Rosmarinic acid showed synergism with α-tocopherol in reducing lipid oxidation of the O/W emulsions, via additional formation of caffeic acid from rosmarinic acid; rosmarinic acid alters the formation of α-tocopherol quinone, and α-tocopherol increases the formation of caffeic acid from rosmarinic acid (Panya et al., 2012). Caffeic acid formation from rosmarinic acid was also shown during the iron-catalyzed oxidation of soybean oil-in-water emulsion with added peppermint extract at pH 6.0 (Kim and Choe, 2018). Esterification of the carboxyl group in polyphenols decreases the solubility in water and alters the interaction with tocopherols. The synergistic effect of rosmarinic acid with α-tocopherol was decreased by esterification, and the eicosyl esters of rosmarinic acid even showed slightly antagonistic interaction with α-tocopherol (Panya et al., 2012).

Synergisms of polyphenols with tocopherols are explained with several mechanisms. Polyphenols protect tocopherols by chelating prooxidative metals (Kurek-Górecka et al., 2014), or forming non-covalent complexes which enable regeneration of tocopherols (Fabre et al., 2015). It was also suggested that co-presence of tocopherols in the emulsion containing herb extracts can shift a major role of polyphenols as antioxidants from scavenging lipid peroxyl radicals to tocopheryl radical scavenging (Kim and Choe, 2019), which can regenerate tocopherols. Regeneration of α-tocopherol by polyphenols via hydrogen donation to the tocopheryl radical was also shown in the rosemary extracts (Fang and Wada, 1993; Wada and Fang, 1992; Wong et al., 1995). The ability of polyphenols to act as a defense agent by donating an electron to tocopherols critically depends on the reduction potentials of their radicals. Polyphenols with a lower redox potential than tocopherols (0.500 V) can donate electrons to tocopheryl radicals to regenerate tocopherols (Choe and Min, 2009). The reduction potential of (+)-catechin, (−)-epicatechin, (−)-epigallocatechin, (−)-epicatechin gallate, (−)-epigallocatechin gallate was reported to be 0.281, 0.277, 0.287, 0.098 ~ 0.146, and 0.104 ~ 0.153 V, respectively (Baranowska et al., 2018), which enables electron donation from these polyphenols to tocopheryl radicals. Instead of protection of polyphenols on tocopherols, tocopherols may protect polyphenols from degradation depending on tocopherol homologs; γ- and δ-tocopherols at 600 mg/kg lowered polyphenol degradation in soybean oil-in-water emulsion with added ethanol extract of peppermint (400 mg/kg), but α-tocopherol increased polyphenol degradation (Kim and Choe, 2019).

On the contrary to the synergistic effects, the antagonistic effects were observed between α-tocopherol and caffeic acid and between α-tocopherol and rosmarinic acid in the AAPH-induced oxidation of linoleic acid emulsions (Peyrat-Maillard et al., 2013). They also suggested that in the co-presence of α-tocopherol, rosmarinic acid acts as a lipid radical scavenger rather than tocopheryl radical scavenger. The above discussion on the interaction between herb polyphenols and tocopherols suggests that tocopherols generally show synergistic antioxidant activities with herb polyphenols, partly depending on their homologs and oxidation environments, and the protection of tocopherols from degradation or their regeneration by polyphenols is an important protection mechanism.

It is true that studies on the interactions of polyphenols derived from herbs with tocopherols from oils are still within the limited area due to diverse environmental factors affecting their activities. Therefore, more research, including kinetics among phenoxyl radicals, lipid radicals, and tocopheryl radicals under various conditions, is required for the highly stable food emulsions with herbs.
