# Correction to: Roles and action mechanisms of herbs added to the emulsion on its lipid oxidation

**DOI:** 10.1007/s10068-020-00807-6

**Published:** 2020-09-05

**Authors:** Eunok Choe

**Affiliations:** Department of Food and Nutrition, Inha University, 100 Inharo, Michuhol-gu, Incheon, 22212 Republic of Korea

## Correction to: Food Sci Biotechnol (2020) 29(9):1165–1179 10.1007/s10068-020-00800-z

The article “Roles and action mechanisms of herbs added to the emulsion on its lipid oxidation”, written by Eunok Choe, was originally published Online First without Open Access. After publication in volume 29, issue 9, page 1165–1179 the author decided to opt for Open Choice and to make the article an Open Access publication. Therefore, the copyright of the article has been changed to © The Author(s) 2020 and the article is forthwith distributed under the terms of the Creative Commons Attribution 4.0 International License (http://creativecommons.org/licenses/by/4.0/), which permits use, duplication, adaptation, distribution and reproduction in any medium or format, as long as you give appropriate credit to the original author(s) and the source, provide a link to the Creative Commons license, and indicate if changes were made.

The original article has been corrected.

